# Advanced electronic consultation between primary care and cardiology: impact of tele-echocardiography and event-based electrocardiographic monitoring

**DOI:** 10.1093/ehjopen/oeaf159

**Published:** 2025-12-06

**Authors:** Juan José Augusto Sánchez Castro, Alejandro Virgos Lamela, Daniel Rey Aldana, Pilar Mazón Ramos, Francisco Gude Sampedro, José Ramón González-Juanatey

**Affiliations:** Primary Care Service, A Estrada Health Centre, Avenida Benito Vigo 110, A Estrada, Pontevedra 36680, Spain; Universidad de Santiago de Compostela, Rúa de San Francisco, s/n, España; Department of Cardiology, University Hospital of Santiago de Compostela; Health Research Institute of Santiago (IDIS); CIBERCV, Choupana s/n, Santiago de Compostela 15706, A Coruña, Spain; Primary Care Service, A Estrada Health Centre, Avenida Benito Vigo 110, A Estrada, Pontevedra 36680, Spain; Department of Cardiology, University Hospital of Santiago de Compostela; Health Research Institute of Santiago (IDIS); CIBERCV, Choupana s/n, Santiago de Compostela 15706, A Coruña, Spain; Concepción Arenal Health Centre, Santiago de Compostela Health Area, Rúa de Santiago León de Caracas 12, Santiago de Compostela 15701, A Coruña, Spain; Department of Cardiology, University Hospital of Santiago de Compostela; Health Research Institute of Santiago (IDIS); CIBERCV, Choupana s/n, Santiago de Compostela 15706, A Coruña, Spain

**Keywords:** e-consultation, Tele-echocardiography, Event-based ECG monitoring, Digital cardiology, Primary care, Healthcare efficiency

## Abstract

Electronic consultation (econsultation) has proven effective in optimizing communication between primary care and cardiology, reducing waiting times and unnecessary face-to-face referrals. This study assessed the impact of incorporating tele-echocardiography and event-based electrocardiographic monitoring (EEM) into an advanced e-consultation model. A prospective observational cohort of 1200 consecutive e-consultations was analysed; in 354 cases, the advanced pathway was activated, including 300 tele-echocardiograms and 54 EEM studies. Remote resolution increased from 38.0% in the traditional model to 54.3% in the advanced model (*P* < 0.01). Diagnostic agreement was high (*κ*=0.85 for tele-echo vs. standard echo; *κ* =0.75 for primary care vs. cardiologist interpretation), with only 6.7% non-interpretable studies and no major adverse events during a mean follow-up of 19 months. This structured implementation of tele-echocardiography and EEM suggests a feasible, scalable, and safe innovation aligned with digital transformation goals in cardiology.

## Introduction

The integrated electronic health record (EHR) has transformed healthcare delivery by enabling shared access to clinical information across care levels. This development has facilitated new forms of ambulatory management, such as electronic consultation (e-consultation) —a professional communication channel that allows a significant proportion of cases to be resolved without face-to-face visits. It has been consolidated as an effective and safe telecare tool, reducing waiting times, optimizing access to specialist care, and decreasing unnecessary referrals.^1–5^

Focused cardiac ultrasound (Focus), understood as a targeted and limited use of echocardiography to complement physical examination, can be performed by non-cardiologist physicians with specific training. Its goal is to supplement clinical evaluation by providing additional information that improves diagnostic accuracy and therapeutic decision-making.^6–8^

The integration of echocardiography and telemedicine has enabled the development of tele-echocardiography in two complementary formats:

1. Real-time tele-echocardiography, in which the specialist supervises or guides image acquisition during the examination.
2. Deferred-review tele-echocardiography, in which the family physician acquires the images and the cardiologist interprets them afterwards.
3. Our model follows the latter approach, which integrates smoothly into routine primary-care practice, offers a more structured and accurate assessment than handheld focused echocardioscopy, and strengthens coordination between Primary Care and Cardiology.^9–11^

In addition, event-based electrocardiographic monitoring (EEM) through mobile devices allows patients to record an electrocardiogram (ECG) at the exact moment of symptom onset, without geographical or temporal limitations, facilitating early arrhythmia detection.^12^

To date, no studies have evaluated the combined impact of tele-echocardiography and EEM within a structured e-consultation programme between primary care (PC) and cardiology. This model, termed advanced e-consultation, maintains direct cardiologist supervision and operates within the institutional electronic health record infrastructure of the *Servizo Galego de Saúde (SERGAS)*, ensuring full integration into clinical workflows.

The primary objective of this study was to assess the impact of incorporating both tools—tele-echocardiography and EEM—on non-face-to-face resolution rates in cardiology e-consultation.

Secondary objectives were to:

- analyse the diagnostic concordance between cardiologist interpretation and that of trained family physicians; and
- identify specific consultation categories in which these technologies provide the greatest incremental value. Tele-echocardiography and event-based ECG monitoring are alternative tools activated according to clinical criteria, not cumulative interventions.

## Materials and methods

### Setting and study design

This was a prospective observational study conducted within the *Servizo Galego de Saúde (SERGAS)*, in the Santiago de Compostela Health Area, between June 2022 and December 2024.

The advanced e-consultation programme was implemented in the Primary-Care Service of A Estrada, which comprises four health centres and 16 family physicians (12 morning-shift and 4 afternoon-shift doctors).

The study was developed jointly with the Cardiology Department of the University Hospital of Santiago de Compostela, both integrated within the same health area.

### Population context

The primary-care service of A Estrada covers a population of 20 260 inhabitants, of whom 18 082 are aged > 14 years. Older adults (≥ 65 years) account for 28.7% (*n* = 5 778) of the total population, underscoring the relevance of cardiovascular disease management in this setting.

### Equipment and resources in primary care

Echocardiographic examinations were performed using an ACUSON NX2 ultrasound system (Siemens Healthiness) equipped with a P4-2 cardiac probe and dedicated cardiovascular software, fully integrated into the institutional EHR.

Event-based electrocardiographic monitoring (EEM) was carried out using Kardia Mobile 6L devices (AliveCor©, USA), which record 1- or 6-lead ECGs at the moment of symptoms, allowing review by the cardiologist.

### Training and accreditation

The family physician responsible for image acquisition held official SEC accreditation in focused cardiac ultrasound, which requires:

- A 26-hour theoretical course (SEC Campus).
- Supervised completion of 100 echocardiographic studies, including a 15-day rotation in the primary-care setting (50 studies) and a 15-day rotation in the Advanced Cardiac Imaging Unit of the Cardiology Department (50 studies).
- The certification process included an initial online practical examination following the theoretical module and a final on-site practical examination organized by the Spanish Society of Cardiology (SEC).

In addition, the operator received specific practical training in the Cardiology Department for application of the approved acquisition protocol and participated in monthly quality-review sessions with the cardiology team.

### Traditional e-consultation model

In the Santiago Health Area, the traditional e-consultation model between primary care and cardiology is based on five core pillars:

1. Universal access as the single referral pathway to cardiology.
2. Timely resolution, with a median response time < 7 days.
3. Professional communication is restricted to physician-to-physician exchange.
4. Security and traceability through automatic registration in the EHR.
5. Focused clinical question, addressing a single problem per consultation.

Each request includes anamnesis, physical findings, and ECG; chest X-ray or routine laboratory results may be added.

The cardiologist may resolve the case remotely or refer the patient for a single-visit face-to-face consultation, following predefined clinical criteria (*Table 1a*).

**Table 1:** Clinical criteria for remote resolution, tele-echocardiography request, and in-person cardiology evaluation within the advanced e-consultation model

Remote Resolution^a^	Tele-echocardiography Request^b^	In-person Evaluation^c^
Dyspnoea of clearly non-cardiac origin	Dyspnoea of possible cardiac origin	Dyspnoea of possible cardiac origin
Chest pain of clearly non-cardiac origin	Pericarditis-like chest pain	Palpitations (*)
Occasional syncope with non-cardiogenic profile	Syncope of unknown origin	Syncope with cardiogenic profile
Isolated extrasystole without structural heart disease	Heart murmurs	Symptomatic bradycardia
Asymptomatic bradycardia	Oedema of possible cardiac origin	Symptomatic second-degree AV block (Mobitz I/II)
Asymptomatic first-degree atrioventricular (AV) block (PR ≥200 Ms)	ECG signs of chamber enlargement or ventricular hypertrophy	Tachyarrhythmias suitable for cardioversion or ablation
Interpretation of test results and treatment adjustment	Complete bundle branch blocks (RBBB/LBBB)	Recurrent ventricular arrhythmias
	First-diagnosed atrial fibrillation (rate control)	Suspected channelopathies
	Suspected heart failure (*de novo*)	Suspected moderate/severe valvular disease
	Cardiomegaly on chest X-ray	Suspected congenital heart disease
	Suspected ischaemic heart disease	Suspected hypertrophic cardiomyopathy
	Suspected infiltrative cardiomyopathy	Suspected inflammatory, infectious, or toxic myocardial disease
	Suspected thoracic aorta disease	Suspected pulmonary hypertension
	Suspected malfunction of intracardiac devices or prosthetic valves	Significant pericardial effusion

Tele-echocardiography or EEM are requested alternatively based on predefined clinical indications.

^a^Criteria for remote resolution. ^b^Indications for tele-echocardiography request. ^c^Criteria for in-person cardiology evaluation. RBBB = right bundle branch block; LBBB = left bundle branch block; EEM = event ECG monitoring.

Advanced e-consultation model Tele-echocardiography or EEM is requested alternatively based on predefined clinical indications. The advanced e-consultation represents an evolution of this system.

In the cardiologist’s initial response, a tele-echocardiography or EEM may be requested when these tests are expected to provide relevant information for remote decision-making.

Indications followed predefined clinical criteria (*Table 1b*):

Once the requested tests were performed and uploaded to the EHR, a new e-consultation was automatically generated for cardiologist review. The cardiologist could then:

- resolve the case remotely, issuing diagnostic and therapeutic recommendations with follow-up in primary care, or
- schedule a single face-to-face consultation for further management.

**Figure 1:**
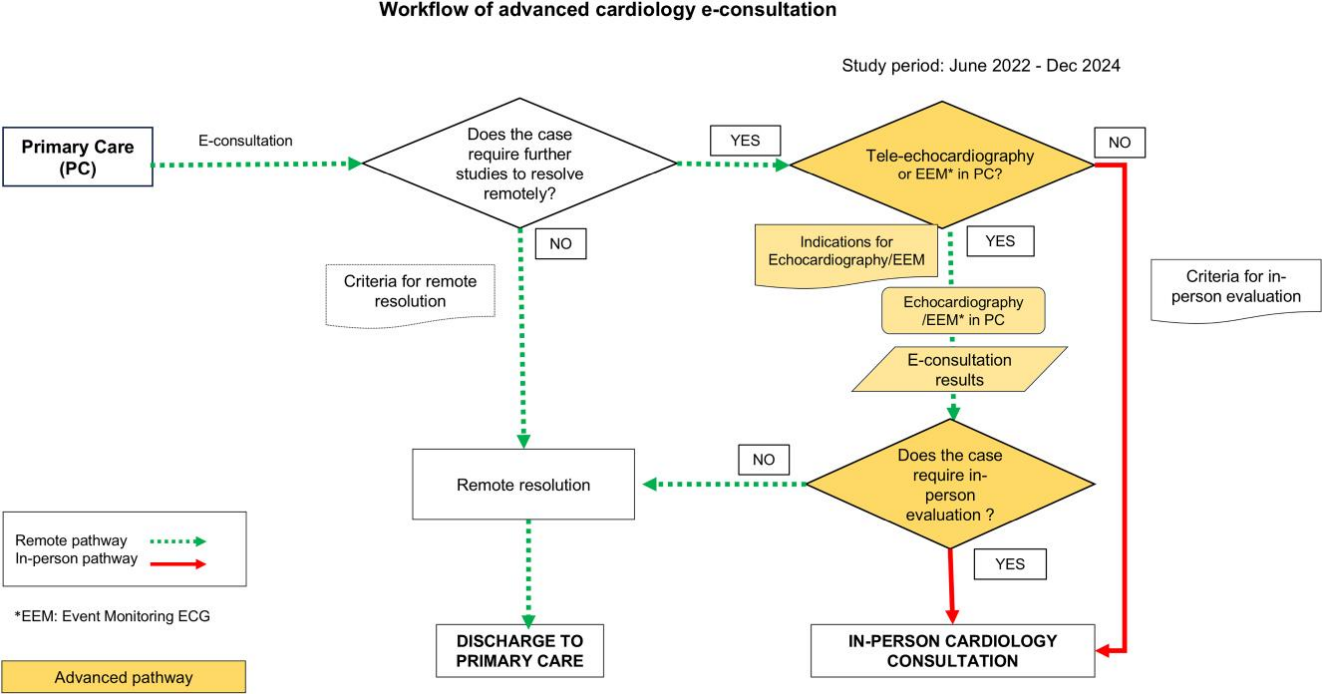
Workflow of advanced cardiology e-consultation, illustrating the decision-making process from primary care through tele-echocardiography and event monitoring ECG (EEM) towards either remote resolution (discharge to primary care) or in-person cardiology consultation. The right-hand branch, outlined in blue, represents the advanced pathway incorporating tele-echocardiography and EEM. Data reflect the study period from June 2022 to December 2024.

The complete workflow is shown in *Figure 1*. Specific criteria for mandatory face-to-face assessment were also established for inconclusive cases, with a protocol-defined maximum delay of within 15 days (*Table 1c*).

### Image acquisition and interpretation

Echocardiographic studies were performed following a structured 22-step protocol, covering three standard windows and nine acquisition planes using M-mode, 2D, colour, pulsed, and continuous Doppler (*Table 2*). All images and video loops were stored within the institutional EHR and subsequently reviewed by the cardiologist without real-time interaction. EEM recordings were analysed in conjunction with the clinical data of the index episode.

**Table 2:** Standardized protocol for echocardiographic image acquisition in primary care

**Parasternal Window**
**1. Parasternal Long Axis View (PLAX)**
1. 2D Mode, Video
2. M-mode: Mitral and Aortic valve, Image
3. Colour Doppler at Aortic/Mitral valve. Video
**2. Parasternal Short Axis View (PSAX)**
**Great Vessels**
4. 2D Mode. Video
5. Pulsed Doppler: Pulmonary valve. Image
6. Continuous Doppler: Pulmonary valve Image
**Mitral Valve**
7. 2D Mode. Video
**Papillary Muscles**
8. 2D Mode. Video
**Apical Window**
**3. Apical Four-Chamber View (AP4C)**
9. 2 D mode. VIDEO
10. Colour Doppler at Mitral and Tricuspid valve. Video
11. Pulsed Doppler at Mitral and Tricuspid valve. Image
12. Continuous Doppler at Mitral and Tricuspid valve. Image
13. M-Mode in lateral Mitral annulus and lateral tricuspid annulus. Image
**4. Apical Five-Chamber View (AP5C)**
14. Colour Doppler in Aortic valve, Video
15. Continuous Doppler in Aortic valve. Image
16. Pulsed Doppler in left ventricular outflow tract. Image
**5. Apical Two-Chamber View (AP2C)**
17. 2D Mode. Video
18. Continuous Doppler in Mitral valve. Image
**6. Apical Three-Chamber View (AP3C)**
19. 2D Mode
20. Colour Doppler in Mitral valve. Video
21. Colour Doppler colour in Aortic valve, Video
**Subcostal Window**
**7. Subcostal View (SC)**
22. M-Mode in inferior cava. Image

All studies were performed following this standardized protocol to ensure diagnostic image quality and reproducibility across operators.

## Variables and statistical analysis

### Variables

We collected:

- Demographic variables: age and sex.
- Clinical variables: reason for consultation.
- Echocardiographic variables: 18 predefined findings assessed by an accredited family physician trained in echocardiography, who classified each as *significant* or *not significant* according to the quantitative thresholds detailed in *Table 3* and the structured checklist described in Supplementary material online, Appendix  *I*.

**Table 3:** Criteria for significant findings in tele-echocardiography

Finding	Criteria
Left ventricular hypertrophy (LVH)	Septal thickness in diastole >12 mm
Left atrial enlargement	Area >25 cm^2^
Right atrial enlargement	Area >25 cm^2^
Left ventricular enlargement	Diastolic diameter (parasternal) > 63 mm
Right ventricular enlargement	Tele-diastolic basal diameter >41 mm
Left ventricular systolic dysfunction	Simpson biplane method <50%
Right ventricular systolic dysfunction	Tricuspid annular plane systolic excursion <17 mm
Type I left ventricular diastolic dysfunction	E/A ratio <0.8
Aortic stenosis	Mean gradient ≥25 mmHg or valve area ≤1.5 cm²
Mitral stenosis	Mean gradient ≥5 mmHg or valve area ≤1.5 cm²
Aortic insufficiency	Total pressure half-time <500
Mitral insufficiency	PISA radius ≥6 mm
Tricuspid insufficiency	PISA radius ≥8 mm
Pulmonary hypertension probability	RV/RA gradient ≥40 mmHg
Significant aortic dilation	Thoracic aorta >4 cm
Inferior vena cava dilation	Diameter >2.5 cm
Pericardial effusion	Significant >1 cm
Pleural effusion	Significant >1 cm

Significant findings were defined based on established echocardiographic reference values. All studies were acquired in primary care and interpreted remotely. PISA, proximal isovelocity surface area.

The structured report was attached to the echocardiographic images and transmitted through the electronic consultation platform for cardiologist review. To validate the procedure, the cardiologist compared the interpretation of 200 consecutive cases with subsequent standard echocardiograms.

All data were recorded in the electronic health record. In addition, adverse events—emergency visits, hospital admissions, and cardiovascular mortality—were monitored during follow-up in patients whose cases were resolved without an in-person consultation.

*Concordance validation*. To validate agreement, a cardiologist compared 200 consecutive tele-echocardiograms with subsequent standard echocardiograms, yielding κ = 0.85 (tele-echo vs. standard echo) and κ = 0.75 (primary-care physician [PCP] vs. cardiologist). Agreement was quantified using Cohen’s kappa (κ); standard errors (SE) were obtained from the asymptotic variance of κ (Fleiss’ method), and two-sided 95% confidence intervals (CI) were calculated as κ ± 1.96×SE (see *Table 5* for cell counts and metrics).

*Outcome definition*. Resolution capacity was defined as the proportion of cases managed without in-person visits.

*Statistical analysis*. Categorical variables are presented as frequencies (%) and continuous variables as mean ± standard deviation. Group comparisons used the *χ*² test, with two-sided *P* < 0.05 considered statistically significant. Analyses were performed using SPSS v22.0 (IBM Corp., Armonk, NY, USA).

### Follow-up and ethical considerations

Clinical follow-up was carried out through the shared EHR linking primary care, emergency, and hospital records. Cases were censored upon transfer outside the health district.

All data were anonymized and processed in compliance with the EU General Data Protection Regulation (2016/679), the Spanish Biomedical Research Act 14/2007, and the Organic Law 3/2018 on Data Protection and Digital Rights.

The Research Ethics Committee of Santiago-Lugo (IDIS/2022/024) approved the protocol and granted waiver of individual informed consent, as the study was observational, based on routine clinical data, and involved no intervention beyond standard care.

## Results

### Resolution capacity

A total of 1200 cardiology e-consultations were managed during the study period. Of these, 456 cases (38%) were resolved electronically without additional tests or face-to-face evaluation by the cardiologist.

**Figure 2:**
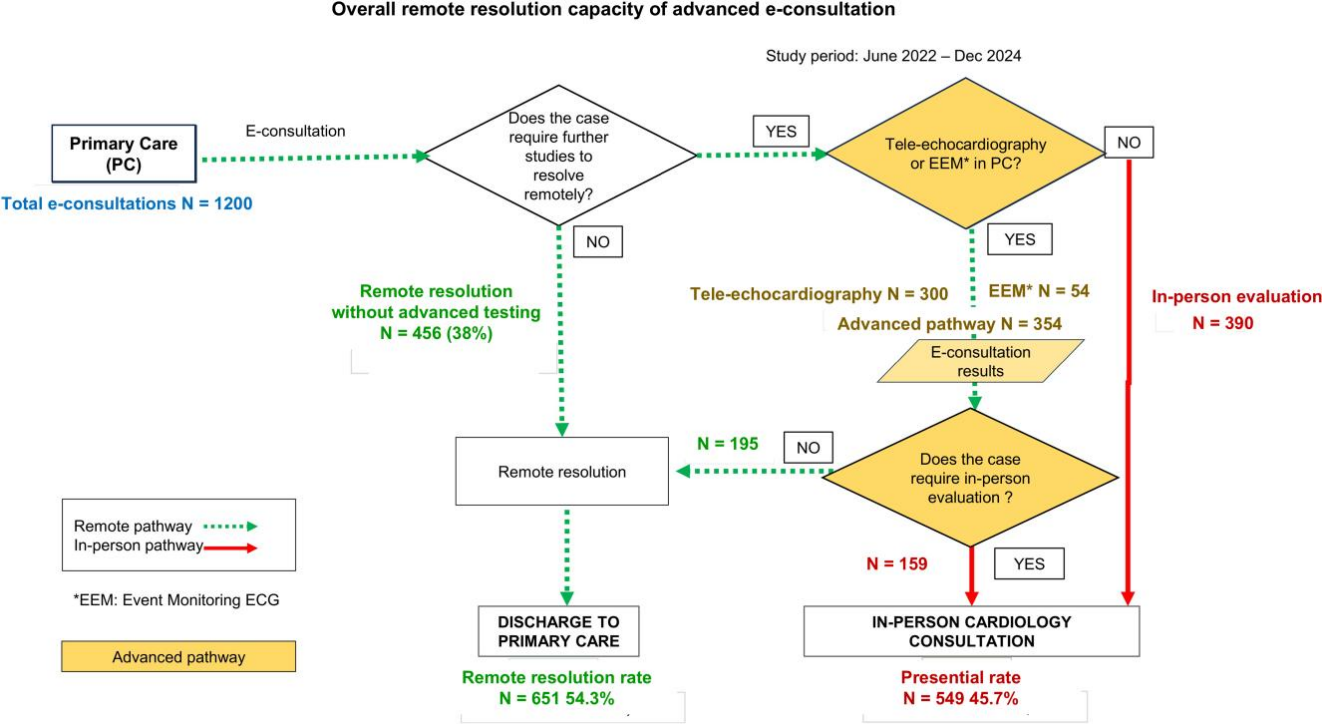
Workflow and outcomes of the advanced e-consultation model between Primary Care and Cardiology. Of 1200 e-consultations, 456 (38%) were resolved without additional tests. Among the 744 cases that required further evaluation, 354 entered the advanced pathway (tele-echocardiography or event-based electrocardiographic monitoring), of which 195 (55%) were resolved remotely, and 159 (45%) required in-person consultation. The remaining 390 underwent other diagnostic studies (not included in the advanced pathway) and were referred directly for in-person cardiology evaluation. Overall, 651 patients (54.3%) were managed without face-to-face visits, and 549 (45.7%) required in-person assessment. Tele-echocardiography or EEM are requested alternatively based on predefined clinical indications.

In 354 cases, the advanced pathway was activated, including 300 tele-echocardiograms and 54 event-based electrocardiographic monitoring (EEM). The overall non-face-to-face resolution rate of the advanced e-consultation was 54.3%, compared with 38% in the traditional model (*P* < 0.01) (*Figure 2*).

**Figure 3:**
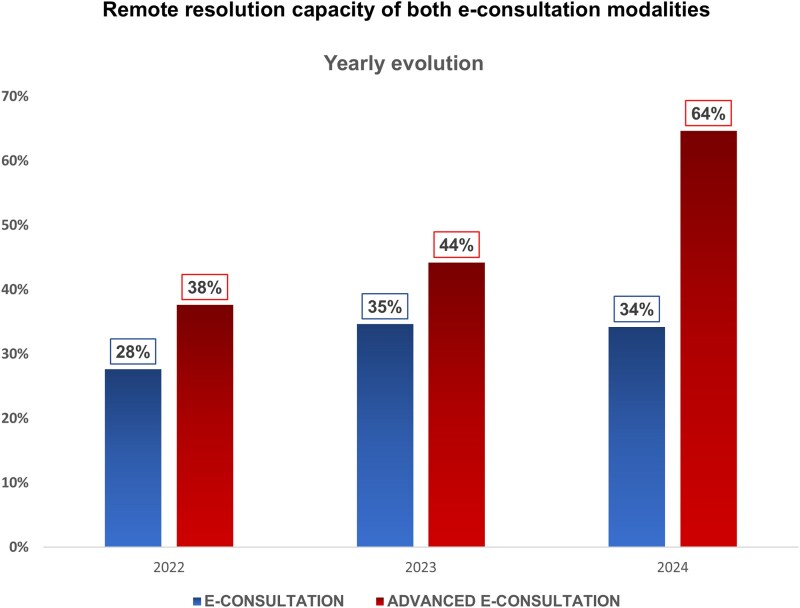
Comparison of remote resolution rates between traditional and advanced e-consultation modalities over the study period (June 2022–December 2024). The advanced model, which integrates tele-echocardiography and event monitoring ECG (EEM), demonstrated a higher overall remote resolution capacity compared with the traditional pathway.

This resolution capacity increased progressively over time: 38% in 2022, 44% in 2023, and 64% in 2024 (*Figure 3*). In total, activation of the advanced pathway allowed the remote discharge of 195 patients without in-person evaluation.

By reason for consultation, tele-echocardiography was particularly useful in cases of heart murmurs and bundle branch blocks, enabling non-face-to-face resolution in 75% and 71% of these cases, respectively, due to the exclusion of significant structural heart disease.

Similarly, EEM avoided in-person visits in 80% of e-consultations related to palpitations (*Table 4*). Ten patients (3.3%) were ultimately referred to the Cardiac Imaging Unit for a standard echocardiogram after face-to-face cardiology evaluation.

**Table 4:** Resolution rate by clinical reason in advanced E-consultation cases

Reason for consultation	*n* (%)	% Women	Age (years)	Remote resolution	Test used
Heart murmurs	101	60.4	68 ± 16	75.2%	Tele-echocardiography
Dyspnoea	47	66.0	78 ± 15	57.4%	Tele-echocardiography
Branch blocks	41	48.8	68 ± 17	70.7%	Tele-echocardiography
Palpitations	54	44.4	44 ± 20	80.0%	EEM^a^
Atrial fibrillation	44	45.5	75 ± 12	56.8%	Tele-echocardiography

Age = Described as mean ± standard deviation

Resolution refers to non-face-to-face management following tele-echocardiography or event ECG monitoring.

^a^In 18 cases, tele-echocardiography was also used.’

### Validation

As part of the validation process, 200 consecutive echocardiographic studies were analysed, comparing the cardiologist’s interpretation of tele-echocardiograms with subsequent standard echocardiograms performed during in-person visits.

The agreement between both diagnostic modalities was high, with a Cohen’s kappa coefficient (κ) of 0.85 (*Table 5*). Additionally, the concordance between the accredited family physician’s interpretations and those of the reference cardiologist was κ = 0.75, with no clinically relevant discrepancies. A total of 6.7% (*n* = 20) of tele-echocardiographic studies were deemed non-interpretable, mainly due to poor acoustic windows or suboptimal image quality.

**Table 5:** Inter- and intraprofessional agreement on significant echocardiographic abnormalities

Significant Abnormality	GP–Cardiologist (GP–CAR)	Cardiologist–Cardiologist (CAR–CAR)
Left ventricular hypertrophy (LVH)	0.62 ± 0.06	0.85 ± 0.05
Left atrial dilation (LA)	0.81 ± 0.05	0.91 ± 0.05
Left ventricular dilation (LV)	0.70 ± 0.14	0.65 ± 0.19
Right atrial dilation (RA)	0.60 ± 0.08	0.96 ± 0.04
Right ventricular dilation (RV)	0.66 ± 0.16	0.80 ± 0.20
Left ventricular systolic dysfunction	0.82 ± 0.09	0.90 ± 0.09
Right ventricular systolic dysfunction	0.66 ± 0.31	1.00 ± 0
Diastolic dysfunction (Type I)	0.76 ± 0.47	0.80 ± 0.06
Aortic stenosis	0.93 ± 0.07	1.00 ± 0
Aortic regurgitation	0.83 ± 0.06	0.74 ± 0.11
Mitral regurgitation	0.88 ± 0.07	0.71 ± 0.15
Tricuspid regurgitation	0.72 ± 0.09	0.94 ± 0.06
Pulmonary hypertension	0.66 ± 0.16	1.00 ± 0
Thoracic aortic dilation	0.77 ± 0.10	0.73 ± 0.13
Dilated inferior vena cava	0.66 ± 0.31	1.00 ± 0
Overall	0.75 ± 0.14	0.88 ± 0.07

Values are expressed as Kappa index ± standard error.

GP–CAR: Agreement between general practitioner and cardiologist. CAR–CAR: Agreement between the same cardiologist reviewing the echocardioscopic study performed in primary care and the echocardiogram performed by themselves in a cardiology setting.

### Safety

During a mean follow-up of 19 ± 4 months, the clinical evolution of the 195 patients discharged remotely without face-to-face evaluation was monitored.

During this period, only:

- three emergency department visits (1.5%), unrelated to the reason for e-consultation, and
- two hospital admissions (1.0%) for cardiovascular causes were recorded.

No cardiovascular deaths or consultations due to clinical worsening related to the advanced e-consultation strategy occurred.

## Discussion

The implementation of the advanced e-consultation model has had a significant organizational impact by structurally integrating tele-echocardiography and event-based ECG monitoring (EEM) within the routine workflow between PC and cardiology.

Compared with the traditional model, this strategy increased non-face-to-face resolution and enabled a larger proportion of cases to be managed directly from PC without requiring hospital referral, consistent with the performance previously reported in the universal e-consultation system in the same health area, where remote resolution ranged between 21% and 30% depending on population profile and clinical complexity.^1,3,6,7,13^ Tele-echocardiography or EEM are requested alternatively based on predefined clinical indications.

This shift contributes to greater system efficiency by optimizing care pathways, reducing the number of in-person cardiology consultations, and supporting continuity of the clinical process. It also reinforces the role of the family physician within a collaborative framework in which the cardiologist retains diagnostic and therapeutic responsibility.

### Diagnostic feasibility and structured supervision

Previous studies have shown that non-cardiologist physicians, after specific training, can acquire and interpret focused echocardiographic images with a high level of agreement with standard echocardiography, supporting the diagnostic feasibility of this approach in primary care.^14–18^ Technical feasibility of remote echocardiography using smartphone-based systems has also been demonstrated.^19^

Our study expands this evidence by demonstrating the feasibility and safety of integrating both tools—tele-echocardiography and EEM—within a structured, cardiologist-supervised e-consultation system.

In this design, activation of advanced testing was performed by the cardiologist, ensuring appropriate clinical indications aligned with guideline-based practice, focused image acquisition, and optimal diagnostic yield.^20^

This supervised model reduces the risk of indiscriminate test use and maintains technical consistency, distinguishing it from decentralized systems or synchronous tele-guidance models relying on less-trained operators.

*Tele-echocardiography and EEM: complementary tools with clinical value*

Tele-echocardiography proved especially useful for the initial assessment of heart murmurs and bundle branch blocks, enabling the exclusion of significant structural heart disease in most cases and avoiding low-yield referrals.

The low proportion of non-interpretable studies (<7%) supports its applicability under real-world conditions and aligns with previous reports confirming its diagnostic reliability and its capacity to reduce costs and unnecessary travel.^21,22^

Progressive improvement in image quality over time likely reflects an accumulated learning effect from continuous training and quality feedback, contributing to the sustainability of the model and reinforcement of operator skills without requiring in-person training.

EEM was particularly valuable for evaluating palpitations and paroxysmal arrhythmias not captured on conventional ECG. The ability to record an ECG at the precise moment of symptom onset enabled clinically relevant rhythm documentation and guided management without resorting to more complex tests such as Holter monitoring or precautionary referrals.^23^

Together, these two tools—integrated into the shared electronic health record (EHR) and managed under cardiologist supervision—enhanced remote diagnostic capability, shortened response times, and improved the overall efficiency of the PC–cardiology interface without compromising patient safety.

### Concordance and safety

The high diagnostic concordance (κ = 0.85) between tele-echocardiography and standard echocardiography, together with the inter-observer agreement between the accredited family physician and the cardiologist (κ = 0.75), supports the robustness of the model. This intra- and inter-observer consistency confirms that image acquisition and interpretation by specifically trained family physicians can be performed reliably when structured supervision and systematic quality control are in place.

During follow-up of the 195 patients discharged without an in-person consultation, only 3 emergency visits (1.5%) and 2 cardiovascular hospitalizations (1.0%) were recorded, with no deaths attributable to the model. This very low event profile supports the clinical safety of the advanced pathway and aligns with previous studies showing that structured e-consultation systems are associated with fewer avoidable complications and more efficient management of cardiovascular disease.^4^

However, certain limitations must be acknowledged. Event-based ECG monitoring depends on patient-reported symptoms and does not capture asymptomatic or nocturnal arrhythmias. Therefore, the findings should be interpreted within the scope of the model and should not be extrapolated to settings requiring continuous monitoring or to populations at higher arrhythmic risk.

### Organizational impact and sustainability

Beyond clinical outcomes, the model provides additional benefits related to the sustainability of the healthcare system. Reducing unnecessary travel helps to lower energy consumption and carbon footprint—an impact not assessed in this study but previously documented in international telemedicine experiences applied to cardiology.^8,11,24^

From a population perspective, the strategy enhances equity by improving access to specialized care in rural areas or regions with a high proportion of older adults, where travel remains a significant barrier.^25,26^

Moreover, the active involvement of primary-care teams in acquiring diagnostic information strengthens professional autonomy and enhances coordination between levels of care, both essential components of patient-centred models of care.

### Limitations and future perspectives

The main limitation of this study is its observational design without a parallel control group, which precludes causal inference. However, comparison with the performance of the traditional e-consultation system provides a natural reference, contextualizing the observed improvements.

Interpretation by a single, non-blinded cardiologist may introduce confirmation bias, although it ensures technical uniformity. External applicability may be limited to settings with accredited operators and integrated EHR systems.

Finally, no formal cost-effectiveness analysis was performed, though the observed reduction in face-to-face referrals suggests potential efficiency gains. Future multicentre studies with a comparative design and formal economic evaluation are warranted to confirm the scalability and sustainability of the model. The progressive incorporation of automated image analysis and artificial intelligence tools may further enhance diagnostic validation, workflow prioritization, and overall applicability within public healthcare systems.

## Conclusions

This study evaluates an advanced e-consultation model between Primary Care and Cardiology that, for the first time in a structured manner, integrates diagnostic tools such as tele-echocardiography and event-based electrocardiographic monitoring.

The incorporation of these technologies into the clinical workflow enhanced non-presential diagnostic capacity, optimized the use of specialist resources, and maintained high standards of diagnostic reliability and patient safety in appropriately selected cases.

The findings support the operational feasibility of the model within primary care and suggest its potential as an effective strategy to improve access, shorten waiting times, and reduce unnecessary referrals—particularly in settings with geographic barriers or high service demand.

Although the model shows evident potential for scalability, successful implementation in other environments will require adequate technological support, specific professional training, and well-defined collaborative structures between levels of care.

Further studies with larger samples and longer follow-up are warranted to evaluate its impact on major clinical outcomes and long-term sustainability. Tele-echocardiography or EEM is requested alternatively based on predefined clinical indications.

### Use of AI tools

Artificial intelligence tools, including large language models, were used to assist in the English translation, language refinement, and formatting of this manuscript. All content was critically reviewed and approved by the authors.

## Supplementary Material

oeaf159_Supplementary_Data

## Data Availability

The datasets generated and/or analysed during the present study are not publicly available due to institutional restrictions, but are available from the corresponding author upon reasonable request.
