# Supplementary material for: Advanced electronic consultation between primary care and cardiology: impact of tele-echocardiography and event-based electrocardiographic monitoring
Source: Eur Heart J Open. 2025 Dec 6;6(1):oeaf159. doi: 10.1093/ehjopen/oeaf159 (PMC12835815; doi:10.1093/ehjopen/oeaf159)
Supplement: oeaf159_Supplementary_Data [file oeaf159_supplementary_data.docx]

**Supplementary appendix I. Supplementary Appendix I. Structured report on echocardiographic findings by family physicians in primary care**

| **No.** | **Finding** | **Code** | **Not Significant** | **Significant** |
| --- | --- | --- | --- | --- |
| 1 | Left ventricular hypertrophy | HVI | ☒ | ☒ |
| 2 | Left atrial dilation | D-AI | ☒ | ☒ |
| 3 | Left ventricular dilation | D-VI | ☒ | ☒ |
| 4 | Right atrial dilation | D-AD | ☒ | ☒ |
| 5 | Right ventricular dilation | D-VD | ☒ | ☒ |
| 6 | Left ventricular systolic dysfunction | DS-VI | ☒ | ☒ |
| 7 | Left ventricular diastolic dysfunction | DD-VI | ☒ | ☒ |
| 8 | Right ventricular systolic dysfunction | DS-VD | ☒ | ☒ |
| 9 | Aortic stenosis | EA | ☒ | ☒ |
| 10 | Aortic regurgitation | IA | ☒ | ☒ |
| 11 | Mitral stenosis | EM | ☒ | ☒ |
| 12 | Mitral regurgitation | IM | ☒ | ☒ |
| 13 | Tricuspid regurgitation | IT | ☒ | ☒ |
| 14 | Suspected pulmonary hypertension | HP | ☒ | ☒ |
| 15 | Thoracic aortic dilation | DAOT | ☒ | ☒ |
| 16 | Dilated inferior vena cava | DVCI | ☒ | ☒ |
| 17 | Pericardial effusion | DPER | ☒ | ☒ |
| 18 | Pleural effusion | DPLE | ☒ | ☒ |

*This checklist was used by family physicians to record relevant findings during asynchronous tele-echocardiography in primary care. Each item could be marked as “not significant” or “significant” based on predefined clinical criteria.*

**Abbreviations:** HVI, left ventricular hypertrophy; D-AI, left atrial dilation; D-AD, right atrial dilation; DS-VI, left ventricular systolic dysfunction; DD-VI, left ventricular diastolic dysfunction; EA, aortic stenosis; IA, aortic regurgitation; EM, mitral stenosis; IM, mitral regurgitation; IT, tricuspid regurgitation; HP, suspected pulmonary hypertension; DAOT, thoracic aortic dilation; DVCI, inferior vena cava dilation; DPER, pericardial effusion; DPLE, pleural effusion.
